# O04 The potential of pyridylpiperazines efflux pump inhibitors to restore antimicrobial activity in MDR *Klebsiella pneumoniae*

**DOI:** 10.1093/jacamr/dlaf230.004

**Published:** 2025-12-04

**Authors:** C Sobieski, V Meurillon, R Vatinel, E Pradel, J Chatagnon, L Lèbre, R Bonnet, L Van Maele, M Flipo, R C Hartkoorn

**Affiliations:** Univ. Lille, CNRS, Inserm, CHU Lille, Institut Pasteur Lille, U1019—UMR 9017—CIIL—Center for Infection and Immunity of Lille, Lille, France; Univ. Lille, Inserm, Institut Pasteur de Lille, U1177-Drugs and Molecules for Living Systems, Lille, France; Univ. Lille, Inserm, Institut Pasteur de Lille, U1177-Drugs and Molecules for Living Systems, Lille, France; Univ. Lille, CNRS, Inserm, CHU Lille, Institut Pasteur Lille, U1019—UMR 9017—CIIL—Center for Infection and Immunity of Lille, Lille, France; Univ. Lille, CNRS, Inserm, CHU Lille, Institut Pasteur Lille, U1019—UMR 9017—CIIL—Center for Infection and Immunity of Lille, Lille, France; Univ. Lille, CNRS, Inserm, CHU Lille, Institut Pasteur Lille, U1019—UMR 9017—CIIL—Center for Infection and Immunity of Lille, Lille, France; Laboratoire de Bactériologie, Faculté de Médecine-Pharmacie, Université d'Auvergne-Clermont-1, Clermont-Ferrand, France; Univ. Lille, CNRS, Inserm, CHU Lille, Institut Pasteur Lille, U1019—UMR 9017—CIIL—Center for Infection and Immunity of Lille, Lille, France; Univ. Lille, Inserm, Institut Pasteur de Lille, U1177-Drugs and Molecules for Living Systems, Lille, France; Univ. Lille, CNRS, Inserm, CHU Lille, Institut Pasteur Lille, U1019—UMR 9017—CIIL—Center for Infection and Immunity of Lille, Lille, France

## Abstract

**Background:**

*Klebsiella pneumoniae* is a leading cause of healthcare-associated infections, particularly affecting immunocompromised patients. The global threat posed by this pathogen has intensified with the emergence and widespread dissemination of MDR strains, notably those producing carbapenemases and ESBLs. With last-resort antibiotics increasingly compromised by antimicrobial resistance (AMR), there is a dire need for novel solutions to fight these priority pathogens. One potential strategy to fight AMR is through the development of efflux pump inhibitors (EPIs) to potentiate the activity of existing antibiotic, and potentially overcome resistance in clinical MDR isolates. In *K. pneumoniae* and *Escherichia coli*, AcrAB-TolC is the dominant antibiotic efflux pump that efficiently extrudes a plethora of antibiotics from bacterial cells. Our laboratory has recently developed a novel class of pyridylpiperazine (PyrPip)-based EPIs that inhibit the AcrAB-TolC efflux system by binding to a newly identified allosteric pocket within the transmembrane domain of AcrB, and thus boosting many antibiotics in *E. coli* and *K. pneumoniae*. In light of the potential of PyrPip EPIs to boost antibiotic activity in susceptible Enterobacterales, it is important to evaluate their impact on clinical MDR *K. pneumoniae* isolates.

**Objectives:**

To evaluate the impact of two optimized PyrPip EPIs, on the antibiotic susceptibility of clinical MDR *K. pneumoniae* isolates, both *in vitro* and *in vivo*.

**Methods:**

49 fully characterized and sequenced clinical *K. pneumoniae* isolates (with a majority being MDR and expressing carbapenemases and/or ESBLs) were selected from the French National Reference Center for Antibiotic Resistance. These clinical isolates were then screened for their susceptibility to 14 diverse antibiotics, both in the absence and in the presence of the two PyrPip-based EPI. The ability of PyrPip-based EPI to overcome antibiotic resistance (or not) was then linked to the bacterial genotype and validated in genetically engineered laboratory strains of *K. pneumoniae*. To confirm that PyrPip-based EPI can overcome resistance in MDR *K. pneumoniae*, the efficacy of selected synergistic combinations was evaluated *in vivo* using *Galleria mellonella* infected with carbapenemase-producing clinical MDR *K. pneumoniae*.

**Results:**

The data generated clearly mapped the impact of PyrPip-based EPI on the antibiotic susceptibility of clinical MDR *K. pneumoniae* isolates. While inhibition of AcrAB-TolC by PyrPip was insufficient to fully overcome resistance in some antibiotic classes, for several others it successfully restored susceptibility to near or even below the clinical breakpoint. Where PyrPip was able to revert antibiotic resistance *in vitro*, these combinations also significantly protected *Galleria mellonella* infected with MDR *K. pneumoniae* and overcome antimicrobial resistance *in vivo*.

**Conclusions:**

This work clearly maps out the magnitude and spectrum of PyrPip mediated antibiotic boosting in critical priority MDR *K. pneumoniae* strains and demonstrated that these EPI are able to revert AMR in *K. pneumoniae* to several antibiotic classes, both *in vitro* and *in vivo*. Together, this data demonstrates that AcrB inhibitors are able to revert resistance in clinical MDR *K. pneumoniae* strains and supports their development to help fight AMR.

